# Informal Caregivers’ Experiences with Performing Telemonitoring in Heart Failure Care at Home—A Qualitative Study

**DOI:** 10.3390/healthcare10071237

**Published:** 2022-07-02

**Authors:** Ina Thon Aamodt, Irene Lie, Edita Lycholip, Anna Strömberg, Tiny Jaarsma, Jelena Celutkiene, Ragnhild Hellesø

**Affiliations:** 1 Centre for Patient-Centered Heart and Lung Research, Department of Cardiothoracic Surgery, Division of Cardiovascular and Pulmonary Diseases, Oslo University Hospital, 0450 Oslo, Norway; irene.lie@ous-hf.no; 2 Department of Masters and Postgraduate Studies, Lovisenberg Diaconal University College, 0456 Oslo, Norway; 3 Department of Health Sciences in Gjøvik, Faculty of Medicine and Health Sciences, Norwegian University of Sciences and Technology, 2815 Gjøvik, Norway; 4 Clinic of Cardiac and Vascular Diseases, Institute of Clinical Medicine, Faculty of Medicine, Vilnius University, 03101 Vilnius, Lithuania; edita.lycholip@santa.lt (E.L.); jelena.celutkiene@santa.lt (J.C.); 5 Clinic of Cardiac and Vascular Diseases, Centre of Cardiology and Angiology, Vilnius University Santaros Clinics, 08661 Vilnius, Lithuania; 6 Department of Health, Medicine and Caring Sciences, Linköping University, 581 83 Linköping, Sweden; anna.stromberg@liu.se (A.S.); tiny.jaarsma@liu.se (T.J.); 7 Department of Cardiology, Linköping University, 581 83 Linköping, Sweden; 8 Institute of Health and Society, Faculty of Medicine, University of Oslo, 0456 Oslo, Norway; ragnhild.helleso@medisin.uio.no

**Keywords:** informal caregiver, self-care, eHealth, telemonitoring, heart failure

## Abstract

Informal caregivers have an important role in caring for family members at home. Supporting persons with a chronic illness such as heart failure (HF) in managing their self-care is reported to be a challenge and telemonitoring has been suggested to be of support. Aim: to explore informal caregivers’ experiences with performing non-invasive telemonitoring to support persons with HF at home for 30 days following hospital discharge in Norway and Lithuania. Methods: A qualitative explorative study of informal caregivers performing non-invasive telemonitoring using lung-impedance measurements and short message service (SMS). Data was collected using semi-structured interviews with informal caregivers of persons with HF in NYHA class III-IV in Norway and Lithuania. Results: Nine interviews were conducted with informal caregivers of persons with HF who performed non-invasive telemonitoring at home. A sequential process of three categories emerged from the data: access to support, towards routinizing, and mastering non-invasive telemonitoring. Conclusion: Informal caregivers performed non-invasive telemonitoring for the first time in this study. Their experiences were of a sequential process that included access to support from health care professionals, establishing a routine together, and access to nurses or physicians in HF care as part of mastering. This study highlights involving informal caregivers and persons with HF together in the implementation and future research of telemonitoring in HF care.

## 1. Introduction

An increased involvement of informal caregivers in caring for their family members is expected due to an aging population and more people living with chronic conditions. It is estimated that family care accounts for about 80% of all care across the European Union (EU) [1]. Home seems to be the place where more complex care will take place in the future, with informal caregivers as an important source of care, e.g., high-, middle-, and low-income countries [2]. Life expectancy and healthy life expectancy at age 65 have variations in high- and middle-income countries, e.g., expectations of healthy years are almost 16 years for Norwegian women and men, whereas for Lithuanians it is 5–6 years for women and men, and a higher life expectancy with activity limitations [2].

Informal caregivers, such as spouses or family members, are important for supporting self-care in persons with heart failure [3] for monitoring changes of physical symptoms at home [4], or for managing these symptoms [5,6]. A caregiver’s burden may impact their ability and willingness to contribute as much as expected from the specialized and community health care services [7,8]. Providers, as informal caregivers of heart failure (HF) patients, may be especially vulnerable, because a chronic condition may last for several years and impacts the daily life of the family as their roles change [9,10]. Moreover, to support HF patients’ self-care at home for monitoring and treating heart failure symptoms may be a challenge for informal caregivers and they report experiencing stress and anxiety [9,10,11].

The informal caregiver living with a person with HF is presented as dyadic HF self-care [12]. The dyadic interventions addressing the management of chronic heart failure is changing from providing face-to-face to telehealth interventions [12]. Moreover, telemedicine has gained increased interest during the pandemic situation in the follow-up of chronic illness at home [13,14] and has opened new expectations of informal caregivers. Informal caregivers of persons with chronic conditions using remote monitoring of vital signs at home expressed a feeling of empowerment and improved care [15]. A study of the active involvement of the informal caregiver performing non-invasive measurements at home using telemonitoring reports findings of improved self-care behavior for HF patients in NYHA class III-IV [4]. Other studies using non-invasive home telemonitoring in HF care present neutral findings on all-cause hospitalizations and all-cause death [16]. Updated HF guidelines suggest considering non-invasive telemonitoring for HF patients to reduce the risk of cardiovascular hospitalizations (CV), HF hospitalizations, and CV death [13]. Informal caregivers performing non-invasive measurement of lung impedance daily at home is sparsely reported [17]. In this study, we therefore add knowledge from informal caregivers involved in using telemonitoring at home to support HF patients after discharge from hospital.

## 2. Objective

The objective of this study is to explore informal caregivers’ experiences with performing non-invasive telemonitoring to support heart failure patients at home for 30 days following hospital discharge in Norway and Lithuania.

## 3. Methods

### 3.1. Design

An explorative design was applied, conducting interviews with informal caregivers who were involved in measurements of lung impedance during 30 days at home following the discharge of the person with HF. This design was chosen because the approach is useful for investigating characteristics of how real life unfolds [18]. The approach accounts for the context, which was of importance to understand the informal caregivers’ perspectives in supporting their family member in the process of using non-invasive telemonitoring. The study was conducted in Norway and Lithuania because the two countries were engaged in a project regarding the potential of using telemonitoring in heart failure patients’ care at home [19,20].

### 3.2. Setting and Telemonitoring Device

**Figure 1:**
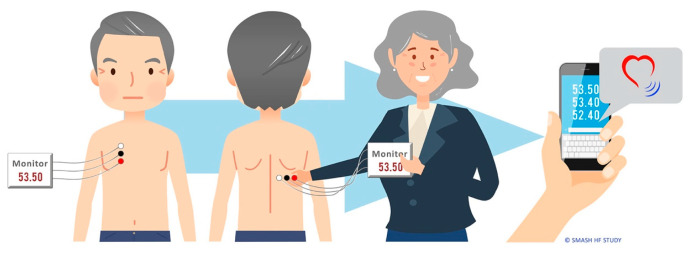
Illustration of how informal caregivers daily placed the cables for measurements of non-invasive lung impedance to support the person with heart failure for 30 days at home. The impedance measurements were transmitted by SMS to the study’s personnel in each country.

Informal caregivers were involved in using a non-invasive CardioSet Edema Guard Monitor (Model 001: CardioSet Medical LTD) to measure lung impedance that may indicate fluid accumulation in the lungs of the person with HF and is reported to start weeks prior to typical HF symptoms [17,21]. The telemonitoring device has not previously been used at home by informal caregivers and persons with HF in their home. It is reported that health care professionals in hospital perform measurements of lung impedance non-invasively with CardioSet Edema Guard Monitor at an HF outpatient clinic [17]. Figure 1 illustrates how the telemonitoring was performed daily by the person with HF and the informal caregiver. First, they had to find the placement and attach three cables on the patient’s chest and three cables on the patient’s back. Secondly, they connected the cables to a device that performed the three measurements of the patient’s lung impedance. Third, after completing the measurements, the results were transmitted using short message service (SMS) to an independent study mobile phone carried by the research nurse in each country as shown in Figure 1.

### 3.3. Participants

A purposive non-probability sample [22] was performed. Informal caregivers of persons with HF discharged from hospital were invited to participate in the study. Inclusion criteria were: aged older than 18 years, fluent in reading Norwegian or Lithuanian, and informal caregiver of a person diagnosed with HF in NYHA class III-IV with marked limitation or inability to carry on any physical activity without discomfort [13]. The informal caregivers were approached by the patient as part of the project [19]. Thereafter, if the informal caregiver wished to participate, information was provided by the researchers orally and in writing before participation. The patients’ criteria are reported in a previous publication focusing on feasibility of non-invasive lung impedance measurements, patients’ symptoms, and changes in self-care during the first month following hospital discharge [19].

### 3.4. Education Session with Using Telemonitoring

The patients and caregivers were educated by a nurse with competency in HF care in each country (ITAa, EL) to perform the non-invasive lung impedance measurements at home. The session started the day after discharge in their home, lasted 1 h, and included demonstration and teach back in preparing and performing measurements at home. Teach back included patients and caregivers performing measurements supervised by the nurse and is recommended in HF guidelines [3,13]. The teach back technique ensured that patients and informal caregivers had understood what had been communicated by the nurse in preparing and performing measurements at home. The session also included oral and written education to all participants, using a handout with heart failure color-coded warnings signs graded as a traffic light, with green representing a stable condition, and recommendations for continuing to monitor symptoms and signs. The handout illustrated gradual changes in symptoms and signs to an unstable condition, illustrated with yellow or orange, and contact information as 911 in case of a severe condition presented by the color red.

### 3.5. Data Collection

Semi-structured interviews were performed using an interview guide with questions to elaborate on the experience of the informal caregivers, supplemented by follow-up and probing questions [23]. The interview guide was developed in English by the research group with clinical and research expertise in HF care and telemonitoring and thereafter translated to Norwegian and to Lithuanian, respectively. The questions were as follows: “Can you describe your experiences with being involved in measurements of lung impedance?”, “Can you elaborate on your daily involvement, did you experience practical or technical issues, concerns, may I kindly ask you to present some examples?”.

The interviews were performed within the days following the final measurement of lung impedance. The setting was chosen by the participants; in general the interview was performed in their home, except one informal caregiver who chose in the hospital because the patient had been re-admitted to hospital. The interviews were performed in Norwegian and Lithuanian, lasted from 30 min to 1 h, were audiotaped and transcribed in the original language after each interview, and thereafter translated to English.

### 3.6. Data Analysis

The transcribed data in English consisted of 74 double-spaced pages for analysis and an inductive approach was used for content analysis inspired by Lindgren, Lundman, and Graneheim [24]. The process started with reading and re-reading the transcribed data and using the objective as a unit of analysis resulting in categories. Meaning units were explored for similar and differences in codes, sub-categories, and categories by four researchers (ITAa, IL, RH, EL). Furthermore, the emerged category was evaluated for additional categories and discussed with other researchers in the study (AS, TJ, JC) to support the credibility with agreement among co-authors. In Table 1, an example of transcribed text, meaning unit, sub-category, and category is presented to ensure the trustworthiness of the content analysis.

### 3.7. Ethical Consideration

This study was conducted in compliance with the principles of the Declaration of Helsinki. All participants were provided with oral and written information about the study prior to participation and signed a written informed consent form. No compensation was provided to the participants in this study. The regional ethics committees in Norway and Lithuania approved the research project (REC no 2014/1890) and Vilnius Regional Ethics Committee (No 158200-15-766-280).

## 4. Results

Nine informal caregivers from Norway and Lithuania participated in interviews concerning their experience of involvement in telemonitoring daily at home for 30 days. The participants were: a mother, wives, and three children who were all adults of both genders. In total, six participants were living together with the person with HF, one lived close to the person, one traveled to the person’s home every day, and one moved the person to their own home for one month. Five of the informal caregivers were working fulltime, one had responsibility for children in preschool and school, and one informal caregiver lived alone. Ethical consideration of their anonymity is the reason for not presenting further demographics of our participants, e.g., informed consent and ethical approval.

Informal caregivers’ experiences of performing non-invasive telemonitoring to support heart failure patients for 30 days following hospital discharge were characterized by three overarching sequential categories. These categories represent a developing process the informal caregivers underwent during the period they performed telemonitoring at home to support the person with heart failure. The category Access to Support indicated their need for practical and technical support when performing measurements at home. Towards Routinizing was the second category, establishing a daily routine and a learning process. Mastering was the third category, informal caregivers experiencing assessing measurements and symptoms of the person with HF.

**Figure 2:**
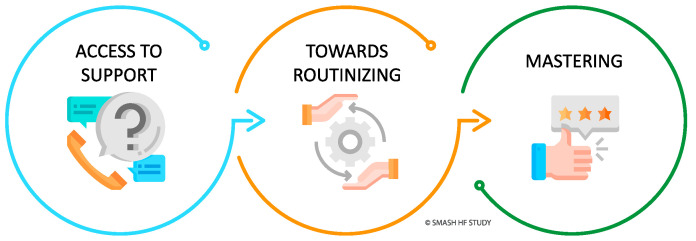
Informal caregivers’ process of access to support, towards routinizing, and mastering performing telemonitoring to support the person with heart failure.

The three categories are illustrated in Figure 2.

In the following, the informal caregivers’ process will be elaborated and presented with citations from the interviews with the nine informal caregivers.

### 4.1. Access to Support

The participants were first-time users of non-invasive telemonitoring; they expressed struggles and uncertainty in the first days, with examples of alarms due to bad connection and in need of technical and practical support. Some had concerns about the potential impact on incorrect lung impedance values. When they approached and got advice from the nurse by telephone, the recommendation could include a small change in the HF patient’s position, e.g., sitting in a more upright position or slightly bending their back for optimal connection of the patches and cables. Other advice from the nurse they found useful were related to how they could improve connection between the patches and the patient’s skin by a rotation of individual patches, additional gel, or changing patches. On some occasions, the nurse decided to visit their home, as illustrated in the following quote:

*“I felt a bit uncertain about the patches and placing them in the right way. The nurse came to our home and confirmed that I did it correct. That was nice. Sometimes I also look at the illustrations, then I am more certain”.*

Participant no. 1 (wife).

The participants did not experience technical problems with low battery alarms during the 30 days of performing non-invasive lung impedance measurements.

### 4.2. Towards Routinizing

The first days of performing lung impedance measurements were exemplified as being a novice, as none of the informal caregivers had previous experience in using telemonitoring at home. Developing a routine in their daily life was expressed by the participants and some suggested to continue after the first month as their learning process had reached a higher level.

*“In the beginning, the first two or three days it was a little bit difficult to locate the correct place for the electrode, but later this procedure went automatically. We were taking the measurements in the morning before I was leaving for work and almost at the same time. When we got used to it, the measurements took less than 10 min”*.

Participant no. 6 (wife).

Some expressed challenges with their own feelings in the beginning, whereas others expressed it was fun to do something new that included daily collaboration and reciprocity in measuring lung impedance and sending the values to the nurse. The value of collaboration was expressed in general to more details of dividing tasks, supporting each during measurements of lung impedance, and access to information about the condition of their family member.

*“We did it together because it is so easy to forget. Suddenly my husband asked, where do I press now. Then I could sit next to him and say press start. Because you do get a bit occupied doing this and being two is a comfort. I get more information as well, about how things are with him, his condition”.*

Participant no. 5 (wife).

To support their husband, son, father, or mother in conducting measurements at home included participants’ descriptions of adjusting to a routine and providing motivational and emotional support to the person with HF in performing non-invasive lung impedance measurements.

*“One thing to mention is that he has been ill for å long period of time and been in and out of hospital, and he found it a bit troublesome. So, we had a little motivation talk. That is how it is sometimes he does not want to take his pills either and is part of his cannot bear it anymore”.*

Participant no. 4 (wife).

### 4.3. Mastering

In general, the participants expressed that they become more confident with the technology and mastering the new routine as less time was spent on practical issues. They felt they were able to focus more on their own creativity in managing daily life. This was illustrated by the participants’ creativity in ensuring the quality of the lung impedance measurements, for example, by taking photos of the person with HF’s chest and back or using a waterproof marker to ensure correct placement and optimal measurements. The measurements of lung impedance were visible to the informal caregiver, the patient, and the nurse; this was expressed as a benefit by informal caregivers. Mastering was expressed by participants, who used changes in lung impedance measurements in combination with HF symptoms and signs that indicated a worsening condition when they discussed their loved one with a cardiologist at the hospital for medical support.

*“When I was talking to his doctor because his weight gained and shortness of breath increased, it was a support to have those measurements. I told him that the measurements from the device were worse. We discussed my husband’s symptoms and measurements. He knew we were in this study and performing measurements. My husband was re-admitted to hospital”*.

Participant no. 4 (wife).

## 5. Discussion

The informal caregivers of persons with HF in NYHA class III-IV performed non-invasive telemonitoring at home for the first time in this study. Our study underlined that, even if they expressed satisfaction of being involved in telemonitoring, efforts were taken to manage their work. This is in line with [25], who use the term “chronic homework” for describing the work the informal caregivers are performing in their homes for persons living with a chronic illness [25]. The informal caregivers made great effort in their daily care.

### 5.1. Ongoing Individualized Support

To be able to perform their work, the participants in our study needed access to individualized and tailored support from health care personnel who hold in depth competency in HF care. The support from health care professionals varied by access through telephone, and sometimes being visited by the nurse gave them confidence when they struggled with the new technology or if changes in the condition of the person with HF occurred.

It is likely that having access to support—as our participants experienced—may be regarded as a strategy to promote autonomous and self-confident caregivers. Confidence and access to care are two of the factors that affect self-care of a person with a chronic illness [5], also present in this study’s findings of informal caregivers performing telemonitoring for the first time. The present study illustrates the need for daily support when using non-invasive telemonitoring at home and encourages a future in HF care to involve support of other persons to those who do not have informal caregivers close to their home for long term use of telemonitoring. However, such practices are not part of usual follow-up in either of the participating countries.

Other studies report that follow-up in telemonitoring interventions in adults with chronic heart failure in the community has variations [26]. It is suggested that future effective strategies in HF care involve artificial intelligence and machine learning [27]. These strategies do not include human interaction with health care professionals in HF care for informal caregivers. Others suggest the human factor to be a key to digital technology and support the need of patient activation as well as access to health care professionals for success [28]. In our study, the informal caregivers were actively involved in using telemonitoring and had access to health care professionals. Recent studies report of the support provided by family members and health care professionals to perform self-care at home [4,29]. The active involvement in using non-invasive telemonitoring is suggested to impact HF patient’s self-care [30] and that the self-care of the person with heart failure improved with the informal caregiver’s active involvement. This may be part of an explanation for the measured improvement of the HF patient’s self-care presented in a previous publication [19]. However, access to competent support must be ensured. We suggest involving informal caregivers as family members in guidelines that consider using telemonitoring [13] and in future research recommendations of telemonitoring in HF care.

### 5.2. Establishing Routines Is Teamwork

The informal caregiver’s process of establishing a new routine in their everyday life involved being a team with the person living with HF. The experience of being a congruent team is considered as a facilitator for the involvement in HF care at home [31]. Establishing a routine by managing HF as a team is reported to be a motivation [32] and motivation is a factor affecting self-care of a person with a chronic illness, as well as habits [5]. Other studies of informal caregivers involved in caring for a person with HF at home report of a burden with feelings of stress, anxiety, acceptance, and optimism based on their health beliefs and closeness of their relationship [32,33]. Dombestein et al., (2020) state that informal caregiving mostly addresses the stress–coping paradigm to reduce the caregiver’s burden [34]. However, they emphasize that it is timely to take a more positive turn when the informal caregiver senses satisfaction, autonomy, and expertise, as goals need to be highlighted. Informal caregivers in this study participated in establishing a habit and expressed being motivated to perform daily measurements as a team when introduced to novel technology and telemonitoring to support the person with HF. To be aware, to interpret, and to recognize HF symptoms is part of self-care monitoring, and self-care is a cornerstone in managing HF [5,6]. Patients hospitalized with acute heart failure are reported to be discharged with persistent congestion with an increase in re-admission and mortality rates [13]. Moreover, heart failure symptoms may or may not reflect changes in the chronic condition [6], and the trajectory of an HF diagnosis can be unpredictable, with changes from a stable phase to episodes of acute decompensation with need of hospital care [35]. Monitoring HF symptoms is important for evaluating the response to treatment and HF stability and is also a challenge when the person with HF has co-morbidities such as chronic obstructive pulmonary disease (COPD), diabetes, chronic kidney disease, or anemia [36,37,38]. We suggest involving informal caregivers and the persons with HF in developing a routine as a team when using telemonitoring.

### 5.3. Becoming an Expert

The informal caregivers in the present study did not have previous experience in using telemonitoring and, through a process with access to support and developing a routine in their daily life, some reached a level of mastering telemonitoring. These participants used their previous experience and skill with HF symptoms when performing measurements of non-invasive lung impedance measurements in assessing the person’s HF condition and contacting health care professionals in HF care at a hospital.

The process towards mastering telemonitoring for participants in this study involved achieving new competencies in using the measurements of lung impedance to support them in detecting, interpreting, and responding to HF symptoms, as well as when to consult an HF specialist in hospital. Even if they experienced it troublesome in the beginning to manage the practical issues related to the technology, they developed new skills beyond previous caring expertise. The proses presented by our participants during the 30 days of performing daily telemonitoring have similarities to the middle range self-care theory that focuses on the individual with a chronic illness and factors affecting self-care as access to care, support from others, experience and skill, motivation, confidence, reflection, and habits [6]. We suggest including informal caregivers, not only the individual with the chronic illness as HF, in the further development of the theory. Our findings illustrate the contributions of informal caregivers as factors that are well known in improving self-care of persons with a chronic condition as HF at home following hospital discharge.

## 6. Conclusions

In this study, informal caregivers’ experiences of performing non-invasive telemonitoring for the first time to support heart failure patients in NYHA class III-IV at home daily for 30 days following hospital discharge is presented. The participants reported a process from initially needing access to support towards routinizing and mastering. This study suggests to actively involve informal caregivers to support persons with chronic heart failure when using telemonitoring at home, as they have knowledge and experience that is important in HF care.

Our study provides knowledge that access to support was important when performing non-invasive measurements of lung impedance to support the person with heart failure in their home.

However, to achieve this level of experience and to feel confident, it became obvious that support included technical and practical support, establishing a routine together, and access to nurses or physicians in HF care as part of mastering the process of performing non-invasive telemonitoring to support heart failure patients at home. We suggest involving informal caregivers and persons with HF together in future research using telemonitoring and in scaling up implementation of telemonitoring in HF self-care.

### Limitations and Strengths

First, the number of participants is a limitation, with nine informal caregivers performing measurements of non-invasive lung impedance at home. A strength in this study is the inclusion of participants from two different countries without previous experience of non-invasive telemonitoring, and in various ages and roles as an informal caregiver and in different phases of their lives [39]. Furthermore, the participants’ decisions to participate may be related to the daily follow-up of their loved one by health care professionals in HF care, as this is not part of usual follow-up in either country. Gender, ethnicity, and cultural differences are other limitations in this sample, with one male, Norwegian, and Lithuanian participants, and is encouraged in future research. However, the findings of informal caregivers’ experiences with involvement in telemonitoring are not transferable to other settings, therefore more knowledge is encouraged. Second, the informal caregivers’ involvement over time is a limitation in this study, as the participants used telemonitoring for one month, and does not include long term involvement of telemonitoring [26]. However, our participants were daily involved for 30 days using telemonitoring and living in Norway, a high-income country funded by public resources, and Lithuania, a part of the Baltic region with a mixed funding system of National Health Insurance Fund and state contribution [40,41]. Third, interviews were transcribed to English from the native language; a potential limitation that was strengthened by researchers from these countries in the different stages of analysis.

## Figures and Tables

**Table 1:** Example of analysis from transcribed text, meaning unit, sub-category, and category.

Transcribed Texts	Meaning Unit	Sub-Category	Category
The first days there were alarms with bad coupling and difficulty with the pads. I sent an SMS to you. Participant no. 3 (wife).	There were alarms with bad coupling and difficulty with the pads the first days. I sent you an SMS.	Practical and technical support	Access to support
Those who are suffering from the disease should come to you and undergo training how to live and what to observe. Family doctors only continue prescription of the medication and do not explain anything. Participant no. 8 (son).	Those suffering from the disease should come to you for training how to live and what to observe. Family doctors only continue prescription and do not explain.	Medical support	
I had to go to him every day to help him to apply electrodes on his back. He decided to do it at 12.00 o’clock. It was the middle of my work. Participant no. 9 (son)	Every day in the middle of my work, I had to go to him and help him apply electrodes on his back.	Adjusting to a new routine	Towards routinizing
At first, I doubted whether the measurements were taken correctly with the electrodes appropriately and in the correct place. Participant no. 7 (daughter). I am managing this better and better all the time, and now it is almost automatic. Participant no. 3 (wife).	First, I doubted taking the measurements correctly and electrodes in the correct place. Now, it is almost automatic. I am managing better all the time.	A learning process	
We have made photos of the chest and the back showing where to place the patches and the sequence of the white, red, and black cable. Participant no. 6 (wife).	We made photos to show placement and sequence of the white, red, and black cable.	Assess measurements	Mastering
During this month we observed my mother’s condition when the measurements were low or very high, e.g., if socks made mark on her ancle or when it was very high, and her condition worsened with nausea and dizziness. Participant no. 9 (daughter).	When the measurements were low or high, we observed my mother’s condition, e.g., if socks made a mark on her ancle, nausea, or dizziness.	Assess symptoms	

## Data Availability

Due to the respondents written informed consent data are not available upon request. We have provided an additional, non-author, point of contact that. interested researchers can get in touch with to secure accurancy regarding our data.
